# Transcriptome sequencing and single-cell sequencing analysis identify GARS1 as a potential prognostic and immunotherapeutic biomarker for multiple cancers, including bladder cancer

**DOI:** 10.3389/fimmu.2023.1169588

**Published:** 2023-06-19

**Authors:** Jianqiang Nie, Taobin Liu, Taotao Mao, Hailang Yang, Wen Deng, Xiaoqiang Liu, Bin Fu

**Affiliations:** ^1^ Department of Urology, The First Affiliated Hospital of Nanchang University, Nanchang, China; ^2^ Department of Anesthesiology, The First Affiliated Hospital of Nanchang University, Nanchang, China; ^3^ Jiangxi Institute of Urology, Nanchang, China

**Keywords:** GARS1, pan-cancer, prognosis biomarkers, immunotherapeutic, bladder cancer

## Abstract

**Background:**

Glycyl-tRNA synthetase 1 (GARS1) belongs to the aminoacyl-tRNA synthetase family, playing a crucial role in protein synthesis. Previous studies have reported a close association between GARS1 and various tumors. However, the role of GARS1 in human cancer prognosis and its impact on immunology remain largely unexplored.

**Methods:**

In this study, we comprehensively analyzed GARS1 expression at the mRNA and protein levels, examined genetic alterations, and assessed its prognostic implications in pan-cancer, with a specific emphasis on the immune landscape. Furthermore, we investigated the functional enrichment of genes related to GARS1 and explored its biological functions using single-cell data. Finally, we conducted cellular experiments to validate the biological significance of GARS1 in bladder cancer cells.

**Results:**

In general, GARS1 expression was significantly upregulated across multiple cancer types, and it demonstrated prognostic value in various cancers. Gene Set Enrichment Analysis (GSEA) revealed the association of GARS1 expression with multiple immune regulatory pathways. Moreover, GARS1 exhibited significant correlations with immune infiltrating cells (such as DC, CD8^+^T cells, Neutrophils, and Macrophages), immune checkpoint genes (CD274, CD276), and immune regulatory factors in tumors. Additionally, we observed that GARS1 could effectively predict the response to anti-PD-L1 therapy. Notably, Ifosfamide, auranofin, DMAPT, and A-1331852 emerged as potential therapeutic agents for GARS1-upregulated tumors. Our experimental findings strongly suggest that GARS1 promotes the proliferation and migration of bladder cancer cells.

**Conclusion:**

GARS1 holds promise as a potential prognostic marker and therapeutic target for pan-cancer immunotherapy, offering valuable insights for the development of more precise and personalized approaches to tumor treatment in the future.

## 1 Introduction

Cancer, as a leading cause of death worldwide, profoundly impacts individuals’ quality of life and poses significant economic challenges for patients (1). Immunotherapy, particularly immune checkpoint blockade therapy, holds immense potential in cancer treatment and has made substantial contributions to the management of various cancer types (2). However, the majority of patients fail to achieve sustained responses, and disease progression eventually occurs in most cases (3). Therefore, there is an urgent need for reliable and effective biomarkers to evaluate the response to immunotherapy.

Aminoacyl-tRNA synthetases (ARS) are among the earliest housekeeping enzymes in the evolutionary history of life (4). They attach specific aminoacyl groups to corresponding tRNA molecules, supplying the necessary amino acids for protein synthesis and ensuring the fidelity of genetic translation. Glycyl-TRNA Synthetase 1 (GARS1, also known as GARS) is a bifunctional member of the ARS family, responsible for charging tRNA in both the cytoplasm and mitochondria (5). It contributes to the pathogenesis of autoimmune diseases and neurological disorders in humans, including Charcot-Marie-Tooth Disease and distal SMA type V (6). Recent studies have demonstrated a close association between GARS1 and multiple tumor types (7–10). For example, urinary GARS1 has shown promise as a novel biomarker for diagnosing uroepithelial carcinoma (7). GARS1 overexpression has been associated with poorer prognosis in lung adenocarcinoma patients (8), while inhibition of GARS1 impedes the growth and colony formation of breast cancer cells (9), and depletion of GARS1 hinders cell proliferation and cell cycle progression in hepatocellular carcinoma (HCC) (10). Hence, GARS1 holds promise as a prognostic and therapeutic biomarker across various cancers.

The role of GARS1 in human cancer prognosis and immunology has been minimally explored to date. In this study, we conducted a comprehensive analysis of GARS1 expression and survival disparities across various cancers, utilizing transcriptome sequencing and single-cell sequencing data. We assessed its attributes in human cancer, encompassing mutations, immunological status, drug sensitivity, and impacts on prognosis. Moreover, it offers valuable insights into the involvement of GARS1 in tumor immunotherapy.

## 2 Methods

### 2.1 Data source

RNA sequencing, somatic mutations, and relevant clinical data were obtained from the USUC portal (http://xena.ucsc.edu/), which includes the TCGA cohort for 33 types of cancer and the GTEx cohort for 31 normal tissues, after removing batch effects. We retrieved the expression profiles of 25 tumor cell lines from the CCLE database (https://portals.broadinstitute.org/ccle/).

### 2.2 Differential expression and survival analysis

We assessed GARS1 expression in human cancer and adjacent normal tissues by integrating the TCGA and GTEx datasets. The visualization was performed using the “ggpubr” R package. Additionally, we evaluated GARS1 expression in various normal tissues using the GTEx dataset. We also assessed its expression in tumor cell lines using the CCLE database (http://ualcan.path.uab.edu/). Protein expression of GARS1 was investigated by utilizing the CPTAC database *via* the UALCAN portal. Furthermore, we obtained immunohistochemistry (IHC) images of the GARS1 protein from the Human Protein Atlas (HPA) database (https://www.proteinatlas.org/).

Four clinical indicators, namely overall survival (OS), progression-free survival (PFS), disease-specific survival (DSS), and disease-free survival (DFS) were employed to assess the association between GARS1 expression and patient prognosis. The analysis utilized the Kaplan-Meier (K-M) method, log-rank test, and Cox proportional hazards model, with the results visualized through forest plots and K-M curves.

### 2.3 Genetic variation analysis

Genetic variation analysis of GARS1 in tumors was performed using the cBioPortal tool (https://www.cbioportal.org/). We assessed the frequency of alterations, mutation data, and copy number alterations (CNA) in GARS1 across various cancers.

### 2.4 Immunological landscape

Initially, we investigated the correlation between GARS1 expression and immune subtypes across various cancer types using the TISIDB portal (http://cis.hku.hk/TISIDB/). Subsequently, we assessed the association between GARS1 and the immune microenvironment within tumors. To determine the tumor purity and evaluate the stromal and immune components of the tumor microenvironment (TME), including stromal scores, immune scores, and estimate scores, we computed ESTIMATE scores for all TCGA tumors using the “ESTIMATE” R package (11). The TIMER database (https://cistrome.shinyapps.io/timer/) was employed to investigate the correlation between GARS1 expression and tumor-infiltrating lymphocytes, such as B cells, CD4^+^T cells, CD8^+^T cells, neutrophils, macrophages, and dendritic cells. Additionally, we collected expression profiles of 47 frequently employed immune checkpoint (ICP) genes extracted from the TCGA cohort and conducted co-expression analysis of ICP genes and GARS1 for each tumor. Moreover, using the TCGA cohort, we examined the association between GARS1 expression and tumor mutational load (TMB), microsatellite instability (MSI), and mismatch repair (MMR) genes (including MLH1, MSH2, MSH6, PMS2, and EPCAM). The “reshape2” and “RColorBrewer” R packages were utilized for this analysis. Furthermore, the correlation between tumor neoantigens and GARS1 expression was investigated through the CMIOP portal (https://www.camoip.net/). Lastly, we obtained expression profiles from the IMvigor210CoreBiologies software package and predicted the relationship between GARS1 and immunotherapy by comparing GARS1 expression differences between the response and non-response groups (12).

### 2.5 Single-cell analysis

The TISCH portal (http://tisch1.comp-genomics.org/) is a single-cell sequencing database specifically designed for studying the tumor microenvironment (13). We utilized this database to investigate the involvement of GARS1 at the single-cell level in the tumor microenvironment across various cancers. Furthermore, CancerSEA (http://biocc.hrbmu.edu.cn/CancerSEA/) is a pioneering database that elucidates various functional states of cancer cells at the single-cell level (14). Using this database, we explored the relationship between GARS1 and 14 functional states in different cancers at the single-cell level.

### 2.6 Drug susceptibility analysis

CellMiner (https://discover.nci.nih.gov/cellminer) is a recently developed tool designed for screening anti-cancer drugs. This tool utilizes a dataset comprising 60 cancer cell lines provided by the National Cancer Institute (NCI) in the United States (15). From the CellMiner online tool, we extracted the GARS1 expression data and drug sensitivity data of the NCI60 panel. Subsequently, the correlation between GARS1 expression and drug sensitivity was predicted and visualized using the “ggplot2” R package.

### 2.7 Gene set enrichment analysis

To perform gene set enrichment analysis, we sorted the samples of each cancer based on their GARS1 expression level. The top 30% of samples were assigned to the high GARS1 group, while the bottom 30% were assigned to the low GARS1 group. Differential expression analysis was conducted using the “limma” R package, considering genes with an adjusted P-value < 0.05 as differentially expressed genes (DEGs). Additionally, we downloaded the Hallmark gene set “h.all.v2023.1.Hs.symbols.gmt,” consisting of 50 gene sets, from the Molecular Signatures Database website (MSigDB, https://www.gsea-msigdb.org/gsea/index.jsp). This gene set was used to calculate the normalized enrichment score (NES) and false discovery rate (FDR) of DEGs in each cancer type. Gene set enrichment analysis was performed using the “clusterProfiler” and “GSVA” R packages.

### 2.8 Biological significance of GARS1 in bladder cancer cells

To validate the biological significance of GARS1, we conducted cell function experiments using bladder cancer cells.

#### 2.8.1 Cell culture and transfection

All human bladder cancer cell lines, including 5637, T24, UC3, BIU, and immortalized human bladder epithelial cells (SV-HUC-1), were obtained from the ATCC cell bank (https://www.atcc.org/, USA). These cell lines were stored in liquid nitrogen tanks or -80°C freezers. Culturing of the cells was performed in a 5% CO_2_ incubator at 37°C. We purchased the small interfering RNA (siRNA) targeting the human gene GARS1 from RIBOBIO (https://www.ribobio.com/, Guangzhou, China). The siRNA had the following sequences:

si-GARS1_1 GATGGAGTATCTTGCCATT
si-GARS1_2 GGCATGGAGTATCTCACAA
si-GARS1_3 GGCAGACCTTCACCTTTAT

UC3 and T24 cells were inoculated in 6-well plates (4~6×10^5^ cells/well) and cultured in a 37°C, 5% CO_2_ incubator. Until cell fusion reached 50~70%, cells were transfected with Lipofectamine 2000 (Invitrogen).

#### 2.8.2 Real-time fluorescence quantitative PCR amplification

Total RNA was extracted from the aforementioned five cell lines, and UC3/T24 cells were transfected with si-GARS1. Reverse transcription was performed using the reverse transcription kit provided by Tiangen (https://www.tiangen.com/) to convert the extracted RNA into complementary DNA (cDNA). Fluorescence expression levels were measured using the NanoDrop ND-800 UV spectrophotometer. Amplification was conducted using the StepOnePlusTM PCR system, and the obtained results were normalized to β-actin. The relative expression levels of the genes were determined using the 2^(-ΔΔCt) method.

#### 2.8.3 Western blot assay

The total protein was extracted from the aforementioned five cell lines and UC3/T24 cells transfected with si-GARS1 using RIPA lysis buffer (Applygen, Beijing, China) containing protease and phosphatase inhibitors. Subsequently, the proteins were incubated overnight at 4°C on a PVDF membrane with the following primary antibodies: anti-β-Actin (1:1000, Abcam, USA) and anti-GARS1 (1:1000, Abcam, USA).

#### 2.8.4 CCK8 assay

Following a 48-hour transfection, cells were seeded in 96-well plates at the specified cell densities (T24: 4×10^3^ cells/well; UC3: 5×10^3^ cells/well), with six wells per group. Subsequently, the cells were incubated at 37°C with 5% CO_2_ and assessed at 24, 48, 72, and 96 hours using the CCK-8 kit (https://www.hanbio.net/, HANBIO).

#### 2.8.5 Wound healing assay

Once the transfected cells in the 6-well plates reached confluence, a scratch was created using a 1000 μl pipette tip. The cells were then washed with PBS and serum-free culture medium and incubated in serum-free medium at 37°C for 24 and 48 hours. Images were captured, and the migration rate was calculated for each group.

#### 2.8.6 Transwell migration assay

The transfected cells were detached and then seeded into the upper chamber of a Transwell system at the designated cell densities (T24: 4×10^4^ cells/well; UC3: 5×10^4^ cells/well) in a 200 μl cell suspension. The lower chamber of a 24-well plate was filled with 600 μl of culture medium containing 20% FBS. After 48 hours of incubation, the cells were fixed with 4% paraformaldehyde at room temperature for 30 minutes and stained with 5.0% crystal violet for another 30 minutes. Subsequently, images were captured, and cell counts were conducted using ImageJ.

### 2.9 Statistical analysis

To assess the statistical significance and compare the expression levels of GARS1 between tumor and normal tissues, either a T-test or a Wilcoxon rank-sum test was employed. Pan-cancer survival analysis was conducted using the Kaplan-Meier method, log-rank test, and Cox proportional hazards regression model. The correlation between two variables was examined using either Spearman’s or Pearson’s test. Gene expression data were normalized through logarithmic transformation, and significance was determined with a p-value threshold of 0.05. These analyses were performed utilizing R software (version 4.1.3), Perl (version 5.32.0.1), and online web tools.

## 3 Results

### 3.1 Overview of GARS1

**Figure 1:**
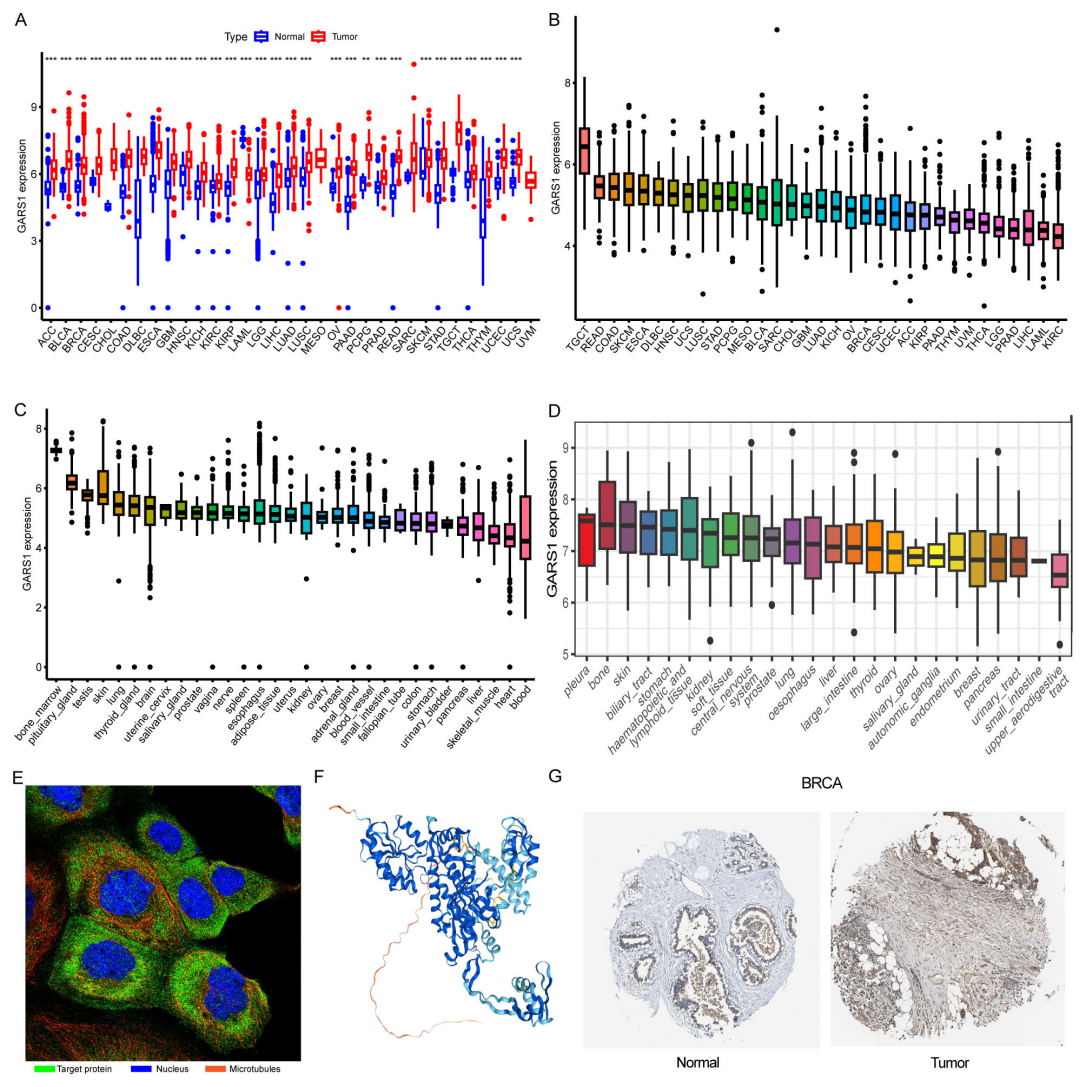
Overview of GARS1. **(A)** Differential expression of GARS1 in tumor and normal tissues. **(B)** GARS1 expression in tumor tissues. **(C)** GARS1 expression in normal tissues. **(D)** GARS1 expression in tumor cell lines. **(E)** Subcellular localization of GARS1. **(F)** Three-dimensional structure of GARS1 protein. **(G)** Immunohistochemical images of GARS1 in normal tissue and tumor tissue of BRCA. **p < 0.01, ***p < 0.001.

**Figure 2:**
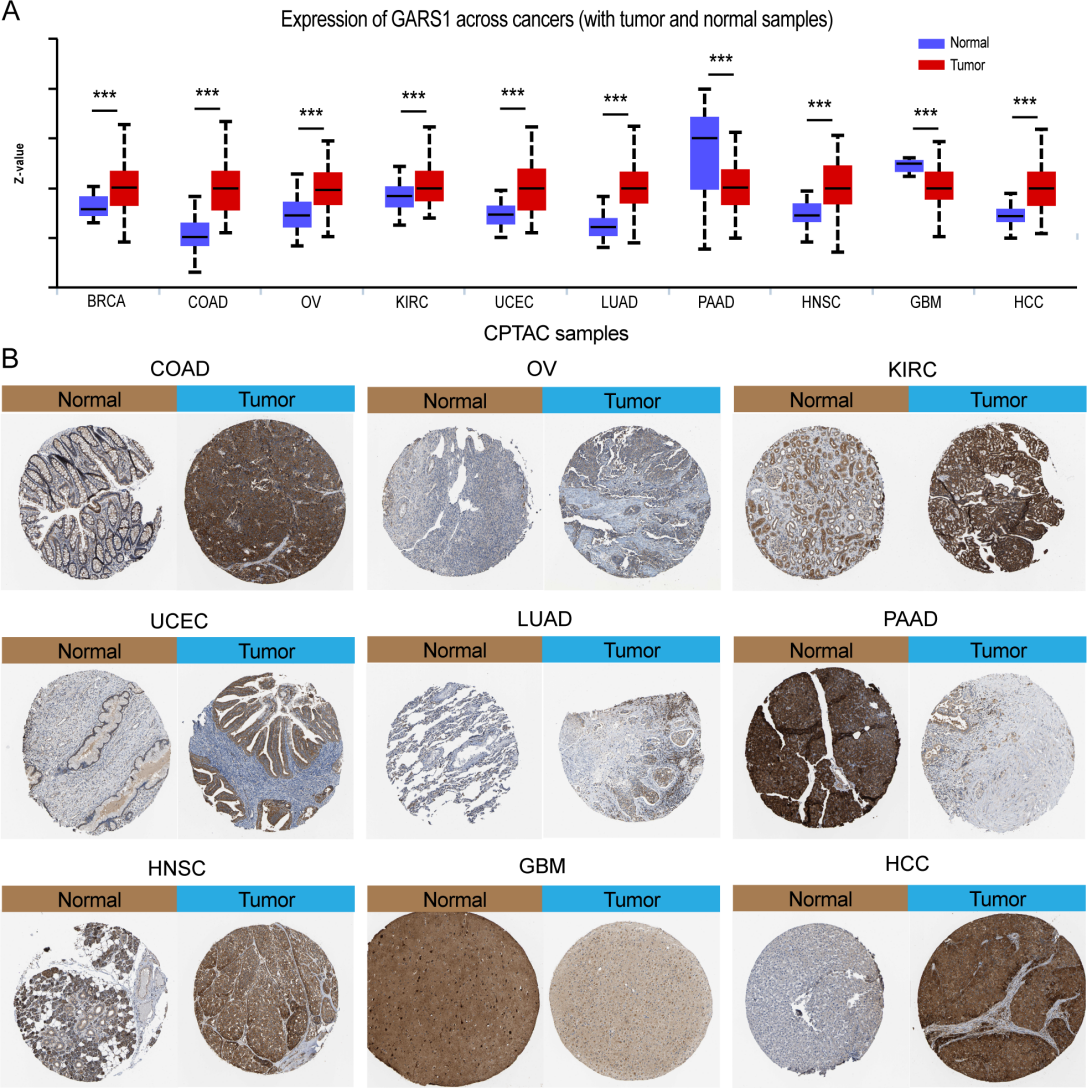
Differential protein expression of GARS1 in tumors and corresponding immunohistochemical images. **(A)** Differential protein expression of GARS1 in multiple cancers. **(B)** Immunohistochemical images of GARS1 in normal and tumor tissues of COAD, OV, KIRC, UCEC, LUAD, PAAD, HNSC, GBM, and HCC. ***p < 0.001.

Initially, we examined the expression levels of GARS1 across multiple cancer types. The findings indicated that, except for MESO and UVM due to the unavailability of adjacent normal tissue data, GARS1 expression was markedly higher in the majority of human cancers (29/33) when compared to adjacent non-cancerous tissues (**Figure 1A**). Notably, TGCT demonstrated the highest expression of GARS1, whereas KIRC exhibited the lowest expression (**Figure 1B**). Subsequently, we investigated the expression levels of GARS1 in normal tissues, revealing its highest expression in bone marrow tissue and lowest expression in blood samples (**Figure 1C**). **Figure 1D** illustrates the relative expression levels of GARS1 in various cell lines, as determined by the CCLE database. The analysis revealed that GARS1 exhibited elevated expression levels in the majority of cancer cell lines, particularly in pleural tumors and bone tumors, while also displaying increased expression in hematopoietic and lymphatic system tumors. Subcellular localization information and a three-dimensional image of the GARS1 protein were acquired by accessing the HPA portal (**Figures 1E, F**). GARS1 exhibited cytoplasmic localization, potentially corresponding to its involvement in the regulation of protein translation. Utilizing the CPTAC database, we observed distinct protein expression patterns of GARS1 between normal and tumor tissues in 10 cancer types, with significantly higher protein levels in tumor tissues except for pancreatic cancer and glioblastoma. Immunohistochemical images sourced from the HPA portal further validated these findings, demonstrating moderate or weak staining in tumor tissues of pancreatic cancer and glioblastoma, contrasting with strong or moderate staining in normal tissues. Conversely, the opposite trend was observed in all other cancer types (**Figures 1G**, **2**). Collectively, these findings indicate the widespread expression of GARS1 in diverse cancer tissues.

### 3.2 GARS1 as a prognostic biomarker in multiple cancers

**Figure 3:**
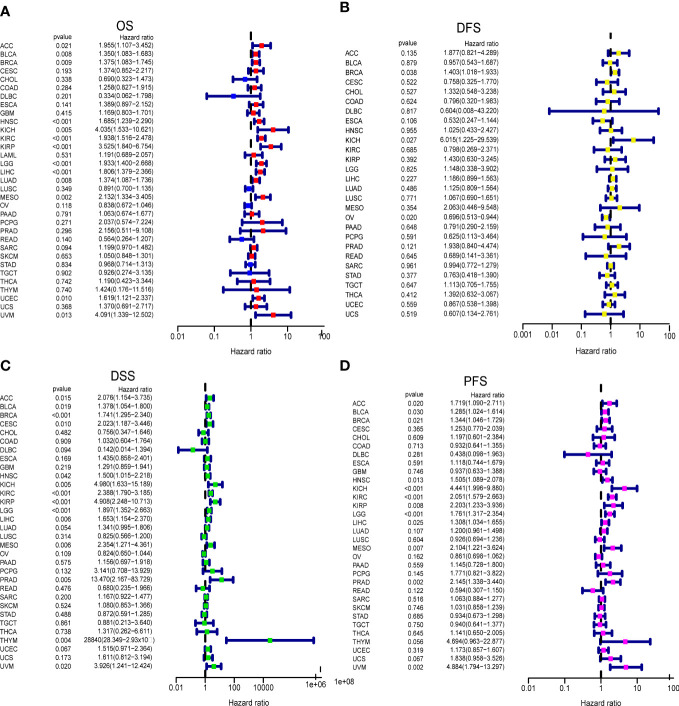
Correlation between GARS1 Expression and Tumor Prognosis using Cox Proportional Hazards Models. **(A)** Correlation between GARS1 expression and overall survival (OS) in different cancer types. **(B)** Correlation between GARS1 expression and disease-free survival (DFS) in different cancer types. **(C)** Correlation between GARS1 expression and disease-specific survival (DSS) in different cancer types. **(D)** Correlation between GARS1 expression and progression-free survival (PFS) in different cancer types.

We analyzed the impact of GARS1 expression on patient prognosis in a pan-cancer cohort. Kaplan-Meier curves demonstrated the significant prognostic significance of GARS1 in ACC, BLCA, BRCA, HNSC, KICH, KIRC, KIRP, LGG, LIHC, LUAD, MESO, SARC, TGCT, UCEC, and UVM (**Supplementary Figure 1**). Forest plots indicated that high expression of GARS1 was associated with unfavorable OS in ACC, BLCA, BRCA, HNSC, KICH, KIRC, KIRP, LGG, LIHC, LUAD, MESO, UCEC, and UVM patients (**Figure 3A**). Patients with higher GARS1 expression exhibited poorer DFS in BRCA and KICH, while it was better in OV (**Figure 3B**). Furthermore, higher expression of GARS1 was associated with worse DSS in ACC, BLCA, BRCA, CESC, HNSC, KICH, KIRC, KIRP, LGG, LIHC, MESO, PRAD, THYM, and UVM (**Figure 3C**). High expression of GARS1 indicated poor PFS in ACC, BLCA, BRCA, HNSC, KICH, KIRC, KIRP, LGG, LIHC, MESO, PRAD, and UVM (**Figure 3D**). Based on these findings, we observed that GARS1 was associated with prognosis in the majority of tumors, indicating its potential as a prognostic biomarker for a wide range of cancers.

### 3.3 Genetic alteration landscape

**Figure 4:**
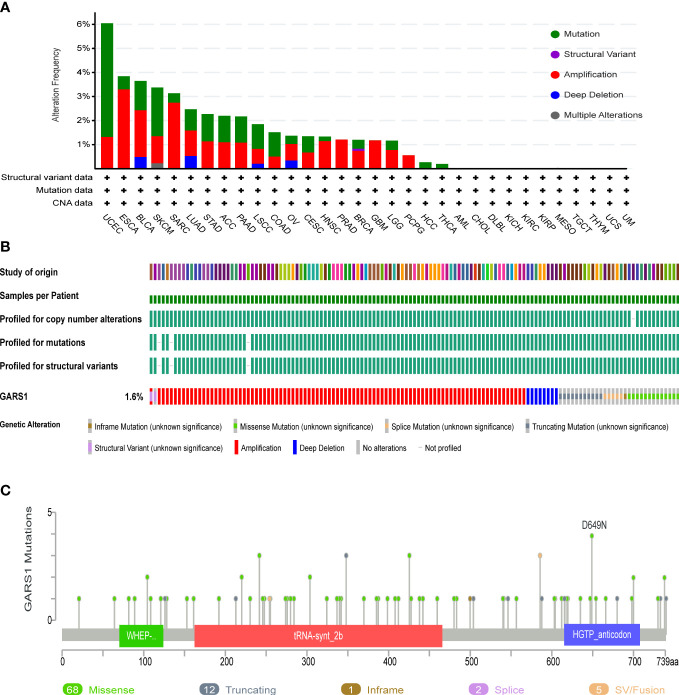
Genetic Alterations in GARS1. **(A)** Frequency of genetic alterations in GARS1 across various cancer types. **(B)** Oncoprint showing the genetic alterations of GARS1 across various cancer types. **(C)** Diagram illustrating the mutations in GARS1 across various cancer types.

Genetic variation information of GARS1 was obtained from the cBioPortal, based on the TCGA cohort (**Figure 4**). GARS1 was found to be altered in 179 (approximately 1.6%) out of 10,967 patients with diverse tumors. UCEC patients exhibited the highest frequency of GARS1 alterations (over 6%), followed by ESCA, BLCA, SKCM, and SARC, with mutation rates exceeding 3%. The main alteration types included amplification and mutation. In total, 88 mutation sites were detected, comprising 68 missense, 12 truncating, 1 nonsense, 2 splicing, and 5 fusion mutations. Among these mutations, D649N was identified as the most common mutation site.

### 3.4 Immunological landscape

**Figure 5:**
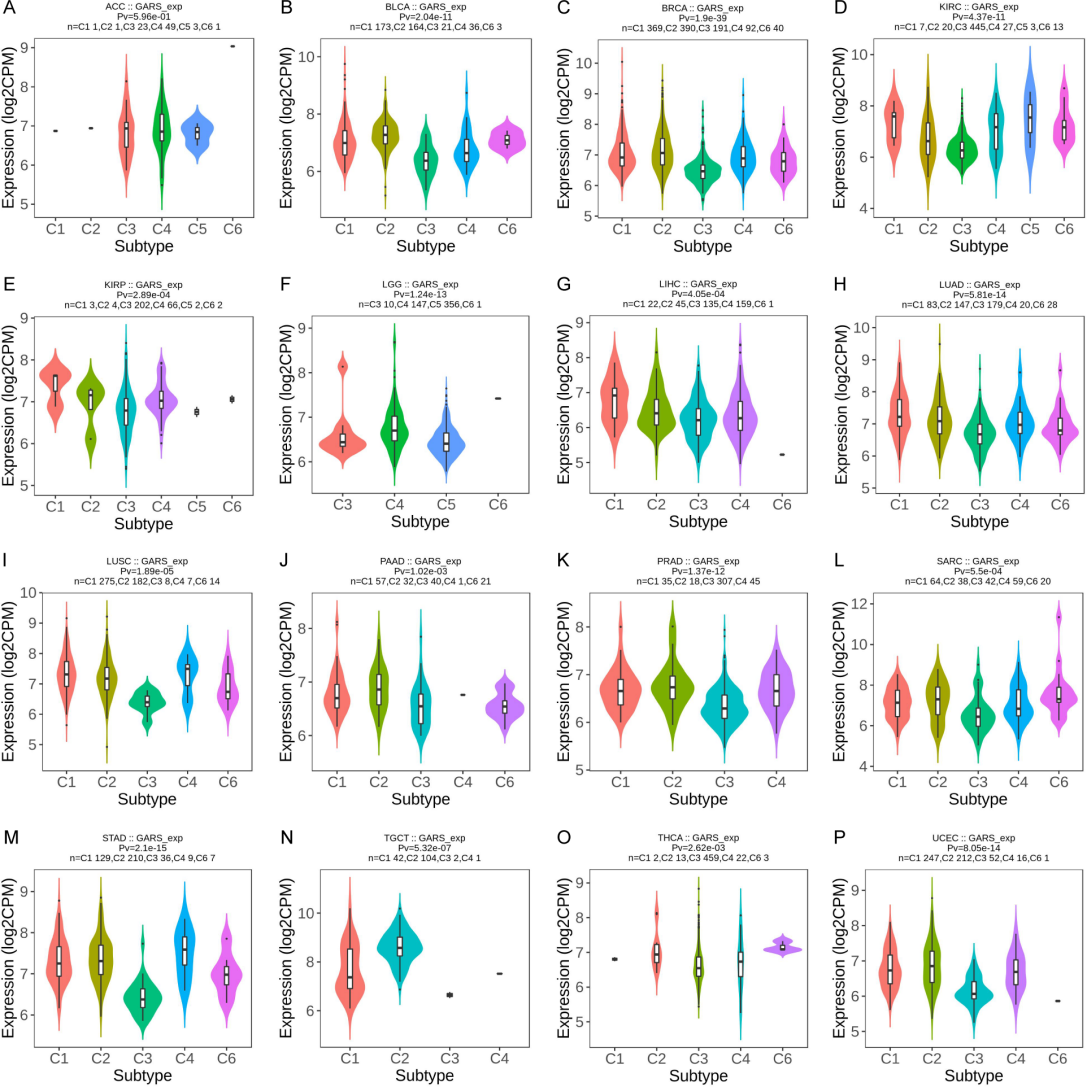
The correlation between GARS1 expression and immune subtypes in various cancer types: ACC **(A)**, BLCA **(B)**, BRCA **(C)**, KIRC **(D)**, KIRP **(E)**, LGG **(F)**, LIHC **(G)**, LUAD **(H)**, LUSC **(I)**, PAAD **(J)**, PRAD **(K)**, SARC **(L)**, STAD **(M)**, TGCT **(N)**, THCA **(O)**, and UCEC **(P)**.

We investigated the correlation between GARS1 expression and immune subtypes in human cancers using the TISIDB website (**Figure 5**). The immune subtypes are categorized into six modules: C1 (wound healing), C2 (IFN-gamma dominant), C3 (inflammatory), C4 (lymphocyte depleted), C5 (immunologically quiet), and C6 (TGF-b dominant). **Figure 5** demonstrates a significant association between GARS1 expression and immune subtypes in 16 specific cancers, including ACC, BLCA, BRCA, KIRC, KIRP, LGG, LIHC, LUAD, LUSC, PAAD, PRAD, SARC, STAD, TGCT, THCA, and UCEC (P<0.05). GARS1 expression varies among immune subtypes across different cancer types. For instance, in the case of LGG, GARS1 exhibited lower expression in the C1 and C2 modules but relatively higher expression in KIRP, indicating distinct roles in cancer progression. Additionally, it was observed that the expression of the C3 immune subtype was generally lower in most tumors, suggesting a limited association between GARS1 and inflammatory immunity.

**Figure 6:**
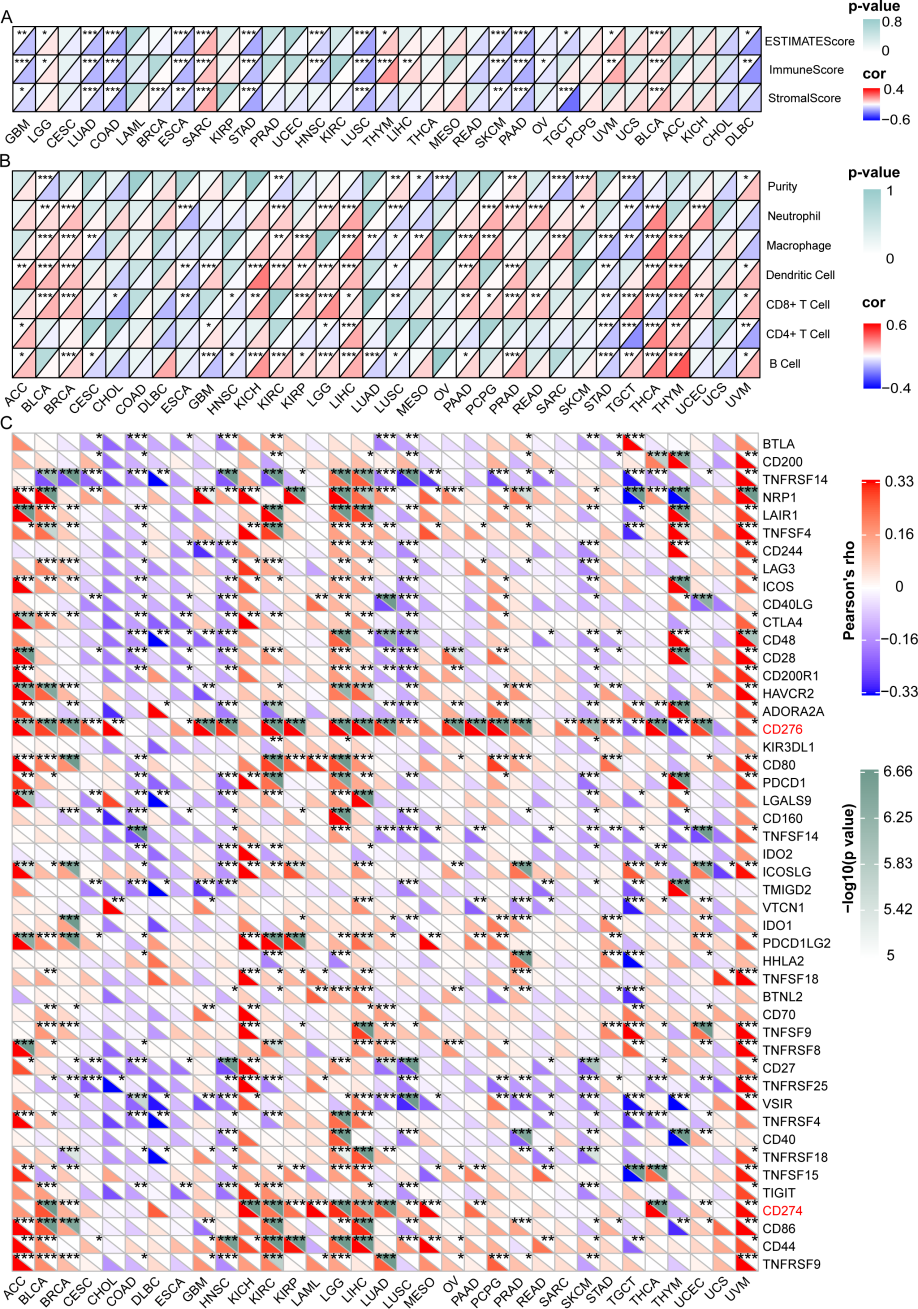
Correlation between GARS1 expression and the tumor immune microenvironment, immune cells, and immune checkpoint genes. **(A)** presents the correlation between GARS1 expression and the tumor immune microenvironment in various cancer types. **(B)** shows the correlation between GARS1 expression and immune cells in different cancer types. **(C)** displays the correlation between GARS1 expression and immune checkpoint genes across various cancer types. *p < 0.05, **p < 0.01, ***p < 0.001.

We then investigated the relationship between GARS1 expression and the tumor immune microenvironment by employing the ESTIMATE algorithm (11). In the majority of tumors, a significant negative correlation was observed between GARS1 expression and immune score as well as estimate score, implying the predominant expression of GARS1 in cancer cells (**Figure 6A**). Conversely, in tumors like BLCA and SARC, a significant positive correlation existed between immune score, stromal score, and GARS1 expression, implying the presence of abundant immune cells during tumor progression.

Data on six immune infiltrating cells from the TIMER database were downloaded for 32 cancer types (excluding LAML), and the correlation between GARS1 expression and immune cell scores was analyzed (**Figure 6B**). It was found that in most tumors, including BLCA, BRCA, KIRC, KIRP, LGG, LIHC, PAAD, PCPG, PRAD, READ, SARC, SKCM, THCA, THYM, and UCEC, GARS1 expression was significantly positively correlated with Neutrophils or Macrophages. However, it was also observed that in these tumors, GARS1 expression displayed a significant positive correlation with dendritic cells (DCs) or CD8^+^T cells.

Additionally, we investigated the relationship between GARS1 and ICP genes across different types of cancer (**Figure 6C**). It is noteworthy that CD276 was significantly positively correlated with GARS1 in 24 types of cancer, while CD274 (PD-1) was significantly positively correlated with GARS1 in 15 types of cancer. The majority of ICP genes exhibited a significant positive correlation with GARS1 in ACC, BLCA, KICH, KIRC, LGG, LIHC, and UVM; however, they displayed a significant negative correlation in COAD, DLBC, HNSC, LUSC, and SKCM. Collectively, these findings highlight the intricate relationship between GARS1 and immune cell activation in the context of anti-cancer immune responses, thereby providing valuable insights for further investigations into GARS1-related tumor immunotherapy.

**Figure 7:**
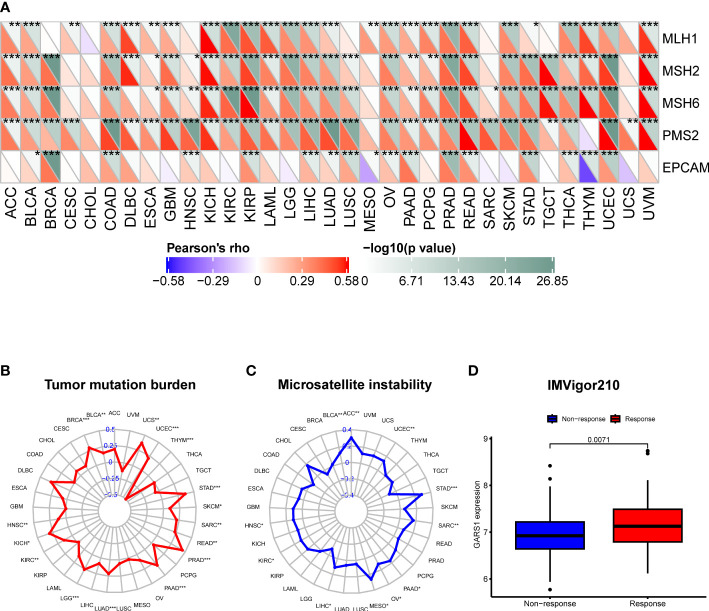
Correlation of GARS1 expression with mismatch repair (MMR), tumor mutational burden (TMB), microsatellite instability (MSI), and immune response. **(A)** A heatmap is presented to illustrate the relationship between GARS1 expression and MMR genes. **(B)** A radar chart depicts the association between GARS1 and TMB. **(C)** A radar chart shows the connection between GARS1 and MSI. **(D)** A box plot demonstrates the correlation between GARS1 expression and immune response within the IMvigor210 cohort. *p < 0.05, **p < 0.01, ***p < 0.001.

TMB, MSI, MMR, and neoantigens are crucial biomarkers for predicting tumor immunotherapy and serve as significant immune regulatory factors (16–18). We examined the association between GARS1 and five MMR genes. The results revealed a significant positive correlation between GARS1 expression and MMR gene expression in the majority of tumors, indicating a potential synergistic effect of GARS1 in tumor repair processes (**Figure 7A**). Furthermore, we conducted an additional analysis to explore the association between GARS1 and TMB. The findings demonstrated a significant positive correlation between GARS1 expression and TMB in numerous tumors, including BLCA, BRCA, HNSC, KICH, KIRC, LGG, LUAD, PAAD, PRAD, READ, SARC, SKCM, STAD, UCEC, and UCS (**Figure 7B**). Additionally, we examined the association between GARS1 expression and tumor MSI. The findings exhibited a significant positive correlation between GARS1 expression and MSI in ACC, BLCA, HNSC, KIRC, LIHC, MESO, OV, PAAD, SARC, STAD, and UCEC (**Figure 7C**). Moreover, utilizing the CMIOP website, we discovered a positive correlation between GARS1 and tumor neoantigens in BRCA, DBLC, LGG, LIHC, LUAD, NSCLC, PAAD, PRAD, SARC, UCEC, and UCS (**Supplementary Figure 2**). The above findings indicate that GARS1 holds substantial predictive value for immunotherapy. IMvigor210 is a study evaluating anti-PD-L1 drugs in the context of advanced urothelial carcinoma (12). Through the analysis of the IMvigor210 cohort, it was determined that GARS1 expression was significantly elevated in the immune response group compared to the non-response group, thereby further substantiating the predictive value of GARS1 in the realm of immunotherapy (**Figure 7D**).

### 3.5 Single-cell analysis of GARS1 in cancers

**Figure 8:**
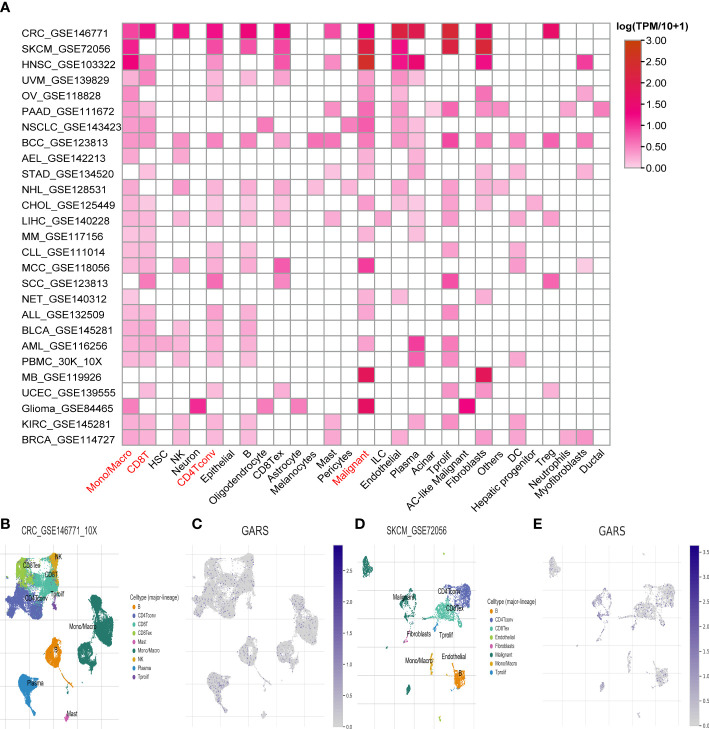
Single-cell analysis of the average expression of GARS1 in different cell types in pan-cancer. **(A)** The average expression of GARS1 is observed in various cell types throughout pan-cancer. **(B, C)** The average expression of GARS1 is assessed in different cell types within the CRC_GSE146771 cohort. **(D, E)** The average expression of GARS1 is examined in various cell types within the SKCM_GSE72056 cohort.

At the single-cell level, a strong correlation between GARS1 expression and the tumor immune microenvironment was observed, as depicted in **Figure 8A**. GARS1 exhibited high expression in the “*CD4Tconv*” “*Mono/Macro*” and “*CD8T*” cell clusters across most tumors, suggesting a robust association between GARS1 and tumor immunity. Furthermore, the “*Malignant*” cell cluster displayed elevated expression of GARS1 in the majority of tumors, indicating its implication in the malignant biological characteristics of tumors. Additionally, UMAP plots of cell clusters and corresponding GARS1 gene scatter plots were depicted for CRC_GSE146771 and SKCM_GSE72056, demonstrating the pronounced expression of GARS1 in immune microenvironment cell types within both tumor types (**Figures 8B–E**).

**Figure 9:**
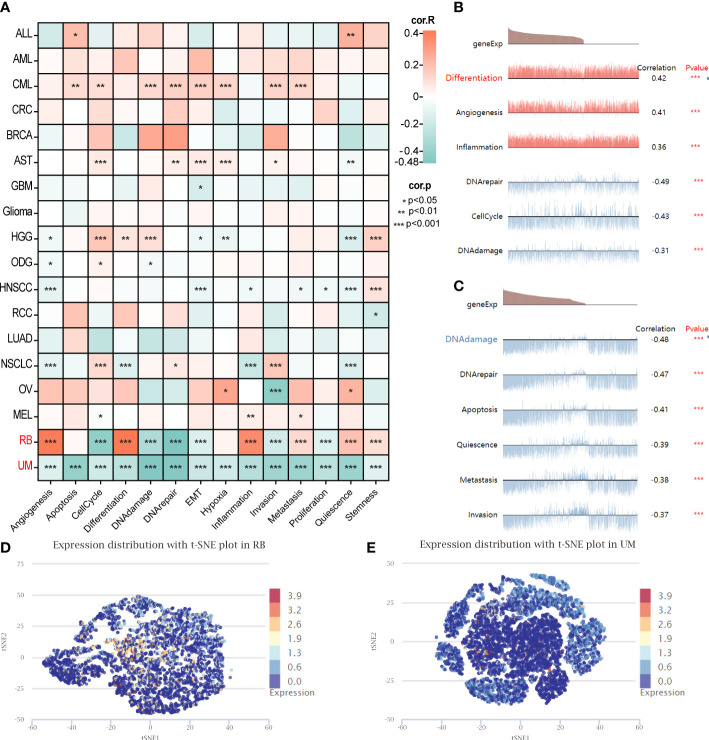
Single-cell analysis illustrating the correlation between GARS1 and 14 functional states in various cancers. **(A)** Correlation between GARS1 and 14 functional states in diverse cancers. **(B)** GARS1 shows significant association with six functional states in Retinoblastoma. **(C)** GARS1 exhibits significant association with six functional states in Uveal Melanoma. **(D)** t-SNE plot depicting the distribution of GARS1 expression in Retinoblastoma. **(E)** t-SNE plot illustrating the distribution of GARS1 expression in Uveal Melanoma. *p < 0.05, **p < 0.01, ***p < 0.001.

Furthermore, an additional analysis was conducted to assess the functional status of GARS1 across diverse tumors. The results revealed significant associations between GARS1 and various cancer-related functions, displaying distinctive patterns among different tumor types (**Figure 9A**). In retinoblastoma (RB), GARS1 exhibited a significant positive correlation with biological functions like *differentiation*, *angiogenesis*, and *inflammation*, while displaying a significant negative correlation with biological functions such as *cell cycle*, *DNA damage*, and *DNA repair* (**Figure 9B**). Furthermore, predominantly negative correlations were observed between GARS1 and diverse biological functions in uveal melanoma (UM) (**Figure 9C**). The UMAP plots of GARS1 in these two cancers similarly demonstrated these implications (**Figures 9D, E**).

### 3.6 Analysis of drug susceptibility related to GARS1

**Figure 10:**
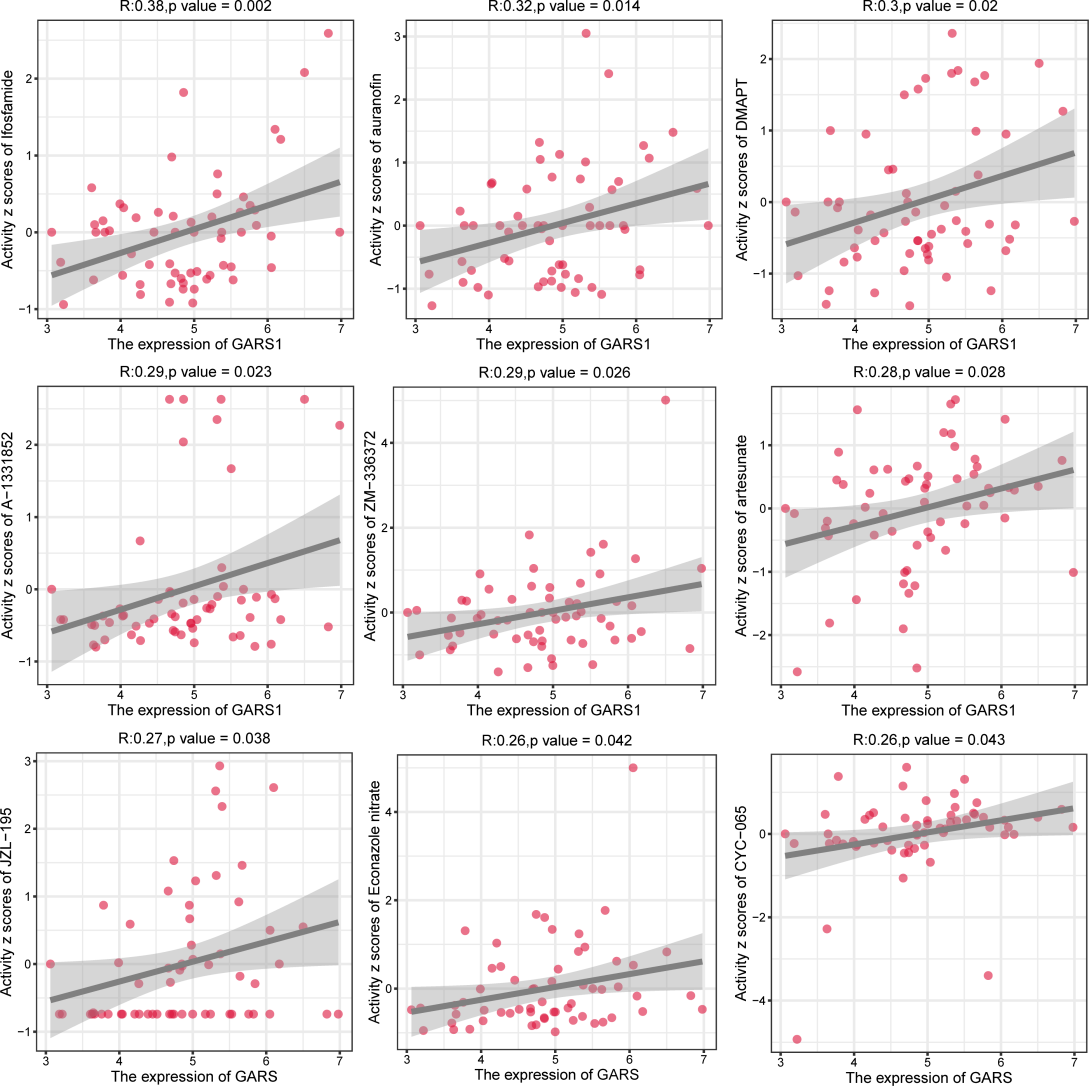
Analysis of drug sensitivity for drugs significantly correlated with GARS1 expression.

In order to provide further guidance for tumor treatment, we investigated drugs that are closely associated with GARS1 (**Supplementary Table 1**). *Ifosfamide*, *auranofin*, *DMAPT*, *A-1331852*, *ZM-336372*, *artesunate*, *JZL-195*, *Econazole nitrate*, and *CYC-065* exhibited a significant positive correlation between their sensitivity and GARS1 expression, indicating their potential use in treating tumors with elevated GARS1 levels (**Figure 10**).

### 3.7 Biological significance of GARS1 in pan-cancer

**Figure 11:**
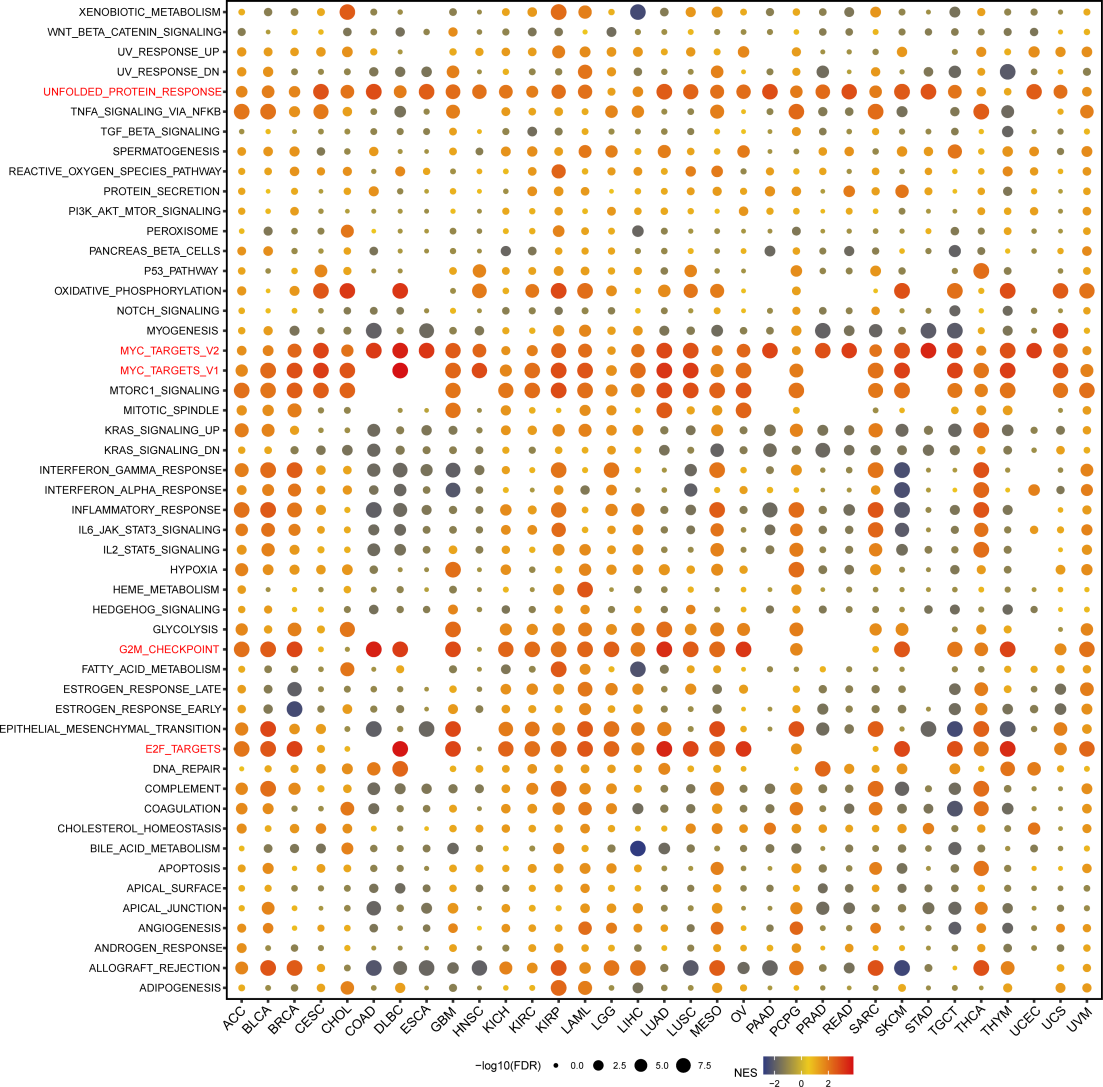
Gene Set Enrichment Analysis of the GARS1 group in pan-cancer.

We performed GSEA analysis to evaluate the biological functions associated with GARS1 (**Figure 11**). Notably, we observed significant enrichment of immune-related pathways, including *MYC_TARGETS_V1*, *MYC_TARGETS_V2*, and *E2F_TARGETS*, in a variety of cancers, especially BLCA, BRCA, DLBC, GBM, KICH, KIRC, KIRP, LAML, LUAD, LUSC, OA, SKCM, TCTG, and THYM. Thus, these findings suggest the potential involvement of GARS1 in the regulation of the tumour immune microenvironment. Moreover, we identified a positive correlation between GARS1 expression and epithelial-mesenchymal transition (EMT) in various cancers, such as BLCA, GBM, LAML, MESO, PCPG, and SARC, suggesting a potential involvement of GARS1 in tumour invasion and migration. Additionally, we found a close association between GARS1 expression and *UNFOLDED_PROTEIN_RESPONSE*, *G2M_CHECKPOINT*, and *OXIDATIVE_PHOSPHORYLATION*. In conclusion, these findings provide evidence supporting the potential involvement of GARS1 in the immune response in cancer.

### 3.8 Effect of GARS1 on the proliferation and migration of bladder cancer cells

**Figure 12:**
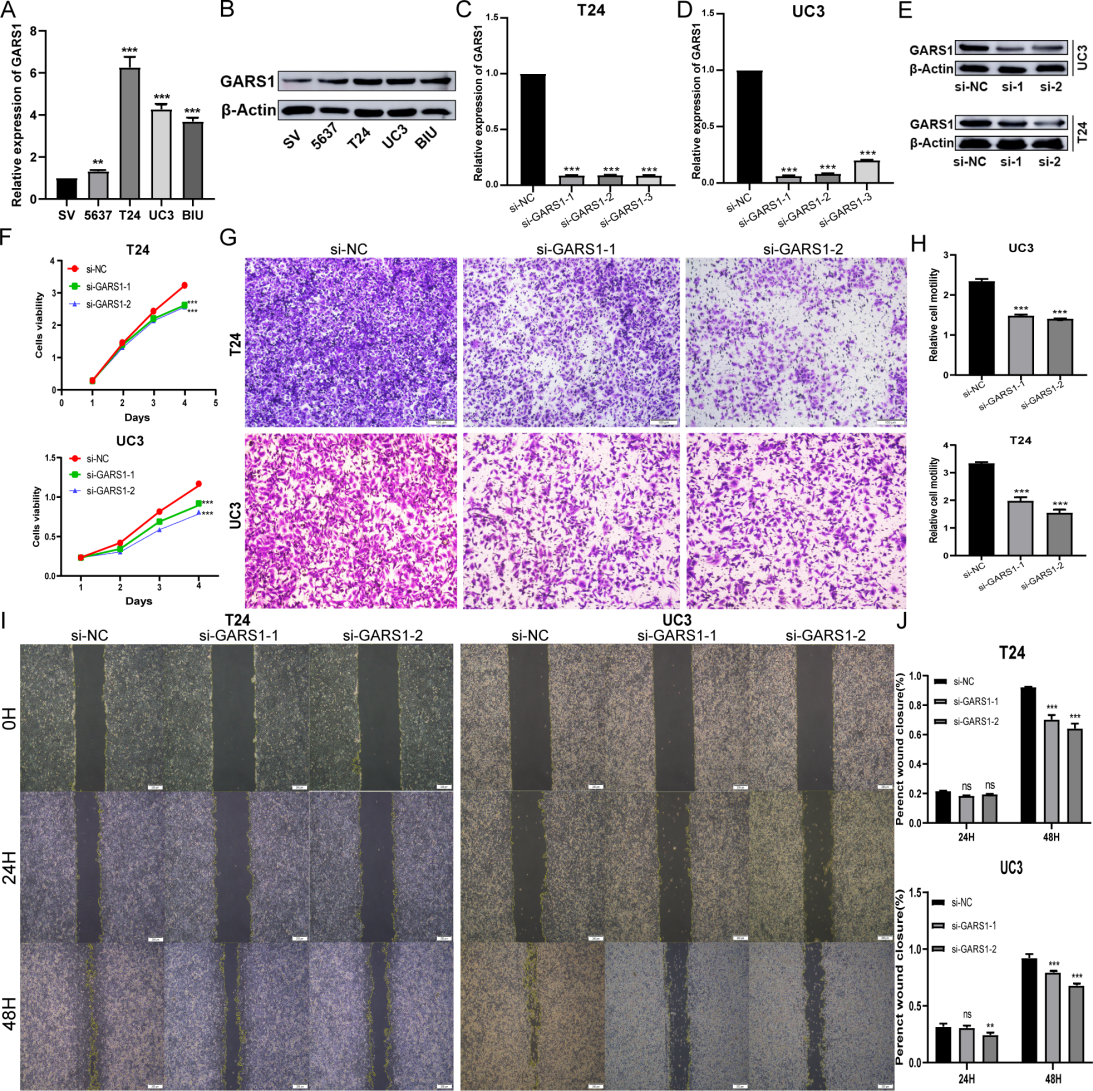
GARS1 promotes the proliferation and migration of bladder cancer cells. **(A, B)** Detection of GARS1 expression in bladder cancer cells and normal uroepithelial cells SV-HC-1 by qRT-PCR **(A)** and Western Blot **(B)**. **(C–E)** Detection of GARS1 knockdown efficiency by siRNA by qRT-PCR **(C, D)** and Western Blot **(E)**. **(F)** CCK-8 test assesses the proliferative capacity of bladder cancer cells after knockdown of GARS1. **(G, H)** Transwell migration test assesses migration of bladder cancer cells after knockdown of GARS1. **(I, J)** Wound Healing test assesses migration of bladder cancer cell UC3 after knockdown of GARS1. ns: *p* ≥ 0.05, **p < 0.01, ***p < 0.001.

Due to the favourable prognostic significance and immune predictive value of GARS1 in bladder cancer, we conducted further investigations into its biological functions in bladder cancer cells. Initially, we validated the high expression of GARS1 in multiple bladder cancer cell lines using qRT-PCR and Western Blot assay (**Figures 12A, B**). UC3 and T24, which demonstrated the highest expression levels, were chosen for subsequent experiments. Subsequently, three GARS1 knockout vectors were constructed and transfected into these two cell lines. The knockout efficiency was confirmed through qRT-PCR and Western Blot assay (**Figures 12C–E**), and Si-GARS1-1 and Si-GARS1-2 with the highest efficiency were selected for subsequent experiments. Subsequently, the CCK-8 assay was utilized to assess changes in cell viability at various time points following transfection in the three groups (Si-NC, Si-GARS1-1, and Si-GARS1-2). The results indicated a substantial decrease in cell proliferation ability 48 hours after GARS1 knockout (**Figure 12F**). Moreover, the Transwell migration assay confirmed a noteworthy reduction in cell migration ability following GARS1 knockout (**Figures 12G, H**). Additionally, the wound healing assay revealed a significant decrease in the healing ability of GARS1 knockout cells (**Figures 12I, J**). In conclusion, GARS1 promotes the proliferation and migration of bladder cancer cells.

## 4 Discussion

Dysregulation of ARS, due to its crucial role in protein synthesis, can contribute to tumor development (4). GARS1, a member of the ARS family, catalyzes the attachment of glycine to tRNA molecules, representing the initial step in protein synthesis within the cytoplasm and mitochondria (19). Previous studies primarily focused on GARS1 have centered around human autoimmune diseases and neuronal disorders. Recently, the significance of GARS1 in the development and progression of diverse tumors has garnered growing attention. For instance, studies have demonstrated its involvement in promoting tumor progression in adrenal cortical carcinoma and urothelial carcinoma (7, 20). In our study, GARS1 exhibited upregulation in the majority of cancer tissues compared to adjacent normal tissues, and its high expression was correlated with a poor prognosis in most tumors. These findings suggest that GARS1, acting as an oncogene, holds potential as a biomarker for pan-cancer diagnosis.

Genetic variations are prevalent factors implicated in carcinogenesis, and the overall mutation rate of GARS1 in TCGA tumors is approximately 1.6%, predominantly involving amplifications and mutations. Moreover, these variations manifest in diverse cancer types, suggesting that GARS1 mutations could contribute to carcinogenesis.

A prior investigation showcased that overexpression of GARS1 *in vitro* enhances macrophage infiltration into HCC cells (10). In normal organs, we observed the highest expression of GARS1 in bone marrow tissue. In cancer cell lines, we noted elevated expression of GARS1 in cell lines originating from lymphatic and hematopoietic system tumors. Additionally, a substantial correlation exists between GARS1 expression and immune subtypes in the majority of cancers, indicating its involvement in immune regulatory processes.

The immune microenvironment is a vital constituent of the tumor microenvironment and plays a pivotal role in tumor immune processes, previously regarded as the “seventh hallmark feature” of tumors (21). Higher stromal and immune scores correspond to lower tumor purity and fewer cancer cells. In our study, GARS1 expression exhibited a negative correlation with stromal and immune scores in the majority of tumors, implying predominant expression by cancer cells. However, in cancers like BLCA and SARC, immune score and stromal score displayed significant positive correlations with GARS1 expression, signifying the presence of abundant immune cells during tumor progression. As a major component of the immune microenvironment, tumor immune lymphocytes have been shown to be an independent predictor of prognosis and immunotherapy efficacy in cancer patients (22). Tumor-associated macrophages and neutrophils possess immune-suppressive abilities, whereas dendritic cells, CD8^+^T cells, CD4^+^T cells, NK cells, etc., are frequent immune-activating cells (23, 24). Dendritic cells’ cross-presentation of antigens is considered the most effective way to activate CD8^+^T cells for the cytotoxic killing of tumor cells (25). Our study discovered a significant positive correlation between GARS1 expression and neutrophils or macrophages in numerous tumors, including BLCA, BRCA, and LIHC, implying that the adverse prognosis associated with GARS1 in tumors may be attributed to immune suppression. Notably, in these tumors, GARS1 exhibited a significant positive correlation with dendritic cells or CD8^+^T cells. This suggests that even during tumor progression, these tumors still retain some immune cytotoxicity. These findings collectively demonstrate an intricate relationship between GARS1 and immune cell activation in anti-cancer immune responses, necessitating additional validation.

Immune checkpoints play a crucial role as targets for immunotherapies (26). Our findings reveal a correlation between GARS1 and immune checkpoint genes across 33 tumor types, suggesting the influence of GARS1 on tumor immunotherapy. Notably, CD274 (PD-1) exhibits a significant positive correlation with GARS1 expression in 15 cancer types, emphasizing the strong association between GARS1 and tumor immunotherapy. PD-1 represents a primary target in clinical immunotherapy. TMB, MSI, MMR, and neoantigens are vital indicators for predicting the efficacy of tumor immunotherapy. Our study demonstrates a significant positive correlation between high GARS1 expression and TMB, MSI, neoantigens, and MMR in the majority of tumors. Tumors characterized by high TMB, MSI, neoantigens, and MMR exhibit improved outcomes following immunotherapy. Additionally, higher expression of GARS1 was observed in the immune response group of the IMvigor210 cohort. This indicates that GARS1 can serve as an effective marker for predicting the efficacy of tumor immunotherapy.

At the single-cell level, we observed a strong correlation between GARS1 and the tumor immune microenvironment. Most tumors exhibited high expression of GARS1 in the cell clusters “*CD4Tconv*”, “*Mono/Macro*”, “*CD8T*”, and “*Malignant*”. These findings suggest a connection between GARS1 and the malignant biological behavior of tumors, further affirming the role of GARS1 in tumor immunity.

Previous studies have demonstrated that GARS1 promotes the cell cycle, migration, and invasion of breast cancer cells by regulating the AKT/mTOR signaling pathway (9). Conversely, depletion of GARS1 hinders the proliferation and cell cycle of HCC cells (10). Our analysis of single-cell data revealed that GARS1 performs distinct functions depending on the tumor type. In Retinoblastoma, GARS1 exhibits a noteworthy positive correlation with biological functions related to *Differentiation*, *Angiogenesis*, *Inflammation*, while showing a significant negative correlation with *CellCycle*, *DNA damage*, *DNA repair*. Conversely, in Uveal Melanoma, GARS1 demonstrates predominantly negative correlations with various biological functions. Additionally, our study revealed that GARS1 is linked to several immune-regulated signaling pathways, including *MYC_TARGETS_V1*, *MYC_TARGETS_V2*, and *E2F_TARGETS*, providing further evidence of the strong connection between GARS1 and tumor immunity.

To further validate the involvement of GARS1 in tumor progression, we specifically chose bladder cancer cells for our experimental investigation. The findings indicated a significant upregulation of GARS1 in bladder cancer cells, and its elevated expression notably stimulated the proliferation and migration of these cells. Consequently, the development of efficacious drugs is imperative. Through data analysis of NCI-60, we observed a substantial positive correlation between drug sensitivity, including *ifosfamide*, *auranofin*, *DMAPT*, and *A-1331852*, and GARS1 expression. This implies that these drugs hold potential as therapeutic choices for tumors exhibiting elevated GARS1 levels. This finding holds crucial reference value for the treatment of GARS1-associated tumors.

This study still has some limitations. First, our analysis relied on sequencing data obtained from open databases, which inherently introduces systematic biases. Second, we lack direct evidence elucidating the impact of GARS1 on tumor prognosis through its involvement in immune infiltration. Third, despite identifying potential drugs that exhibit high correlations with GARS1 using the CellMiner database, we are unable to establish direct interactions between GARS1 and the constituents of these drugs, and the underlying mechanisms of action remain unknown. Therefore, comprehensive experiments are necessary to facilitate clinical application and mechanistic research.

In conclusion, GARS1 is extensively upregulated in cancer and possesses the capacity to predict prognosis across diverse types of cancer. Features such as TMB, MSI, MMR, and neoantigens are strongly correlated with GARS1 in numerous tumor types. GARS1 is intricately connected to tumor immunity. Furthermore, experimental evidence substantiates that GARS1 enhances the proliferation and migration of bladder cancer cells. We have identified a group of drugs that display high sensitivity to GARS1. These findings contribute to the elucidation of GARS1 as a potential prognostic marker and a target for tumor immunotherapy, offering valuable insights for future advancements in precise and personalized cancer treatments.

## Data availability statement

The datasets presented in this study can be found in online repositories. The names of the repository/repositories and accession number(s) can be found in the article/**Supplementary Material**.

## Author contributions

JN and TL designed and carried out the research study. TM and HY contributed to the analysis tools and analyzed the data for the study. JN wrote the manuscript. BF, XL, and WD evaluated and revised the manuscript. All authors contributed to the article and approved the submitted version.

## Supplementary material

Supplementary Figure 1 The correlation between GARS1 expression and tumor prognosis using the Kaplan-Meier method.

Supplementary Figure 2 The correlation between GARS1 expression and neoantigens in pan-cancer.

Supplementary Table 1 Analysis of Drug Susceptibility Related to GARS1.

## Glossary

ACC: Adrenocortical carcinoma
ALL: Acute lymphocytic leukemia
AML: Acute myelocytic leukemia
AST: Astrocytoma
BLCA: Bladder Urothelial Carcinoma
BRCA: Breast invasive carcinoma
CESC: Cervical squamous cell carcinoma and endocervical adenocarcinoma
CHOL: Cholangiocarcinoma
CML: Chronic myelocytic leukemia
COAD: Colon adenocarcinoma
CRC: Colorectal cancer
DLBC: Lymphoid Neoplasm Diffuse Large B-cell Lymphoma
ESCA: Esophageal carcinoma
GBM: Glioblastoma multiforme
HNSC/HNSCC: Head and Neck squamous cell carcinoma
KICH: Kidney Chromophobe
KIRC: Kidney renal clear cell carcinoma
KIRP: Kidney renal papillary cell carcinoma
LAML: Acute Myeloid Leukemia
LGG: Brain Lower Grade Glioma
LIHC/HCC: Liver hepatocellular carcinoma
LUAD: Lung adenocarcinoma
LUSC: Lung squamous cell carcinoma
MEL: Melanoma
MESO: Mesothelioma
NSCLC: Non-small cell lung cancer
ODG: Oligodendroglioma
OV: Ovarian serous cystadenocarcinoma
PAAD: Pancreatic adenocarcinoma
PCPG: Pheochromocytoma and Paraganglioma
PRAD: Prostate adenocarcinoma
RB: Retinoblastoma
RCC: Renal clear carcinoma
READ: Rectum adenocarcinoma
SARC: Sarcoma
SKCM: Skin Cutaneous Melanoma
STAD: Stomach adenocarcinoma
TGCT: Testicular Germ Cell Tumors
THCA: Thyroid carcinoma
THYM: Thymoma
UCEC: Uterine Corpus Endometrial Carcinoma
UCS: Uterine Carcinosarcoma
UVM/UM: Uveal Melanoma.
