# Supplementary figures and images for: Transcriptome sequencing and single-cell sequencing analysis identify GARS1 as a potential prognostic and immunotherapeutic biomarker for multiple cancers, including bladder cancer
Source: Front Immunol. 2023 Jun 19;14:1169588. doi: 10.3389/fimmu.2023.1169588 (PMC10315539; doi:10.3389/fimmu.2023.1169588)

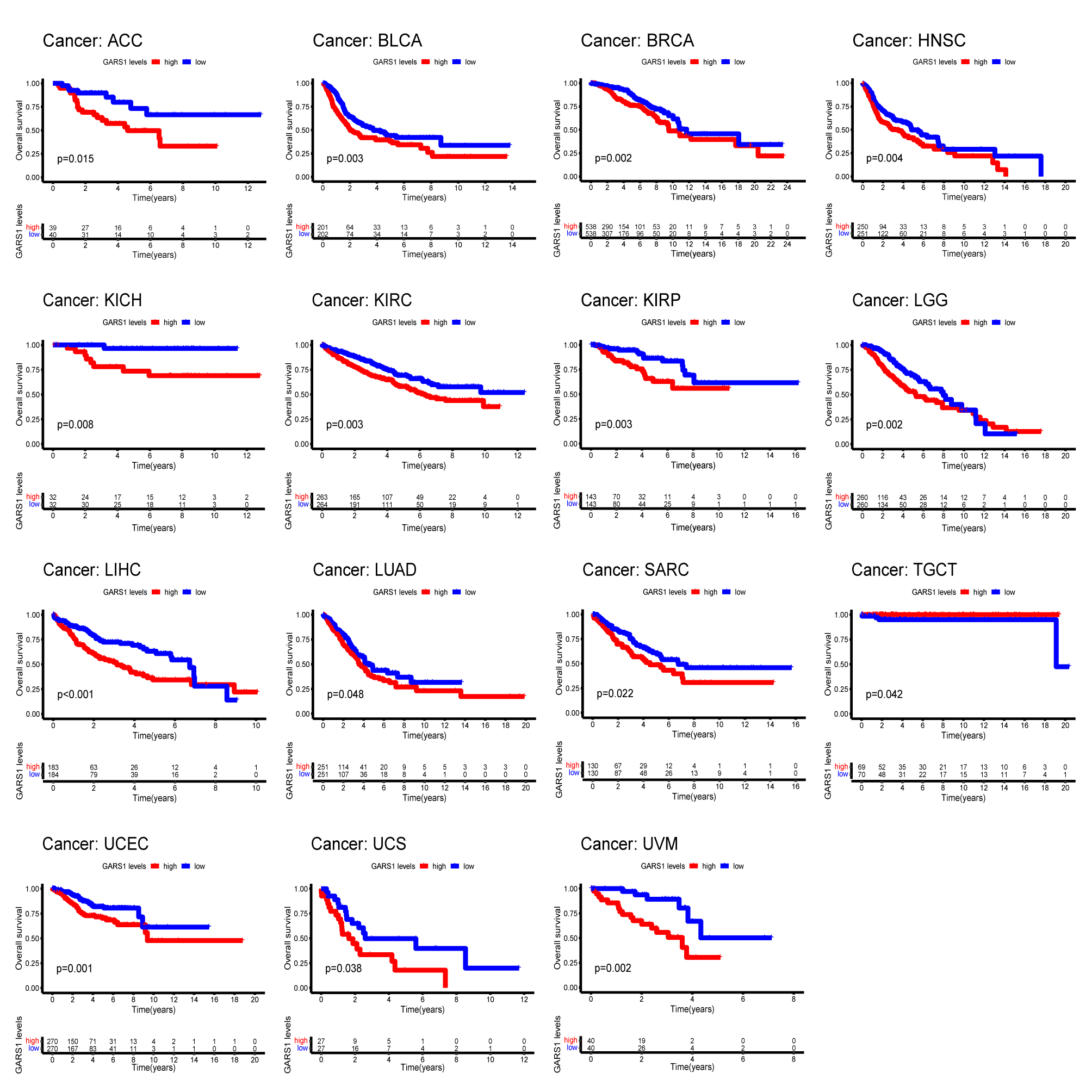

Supplement: Supplementary Figure 1 — The correlation between GARS1 expression and tumor prognosis using the Kaplan-Meier method. [file Image_1.tif]

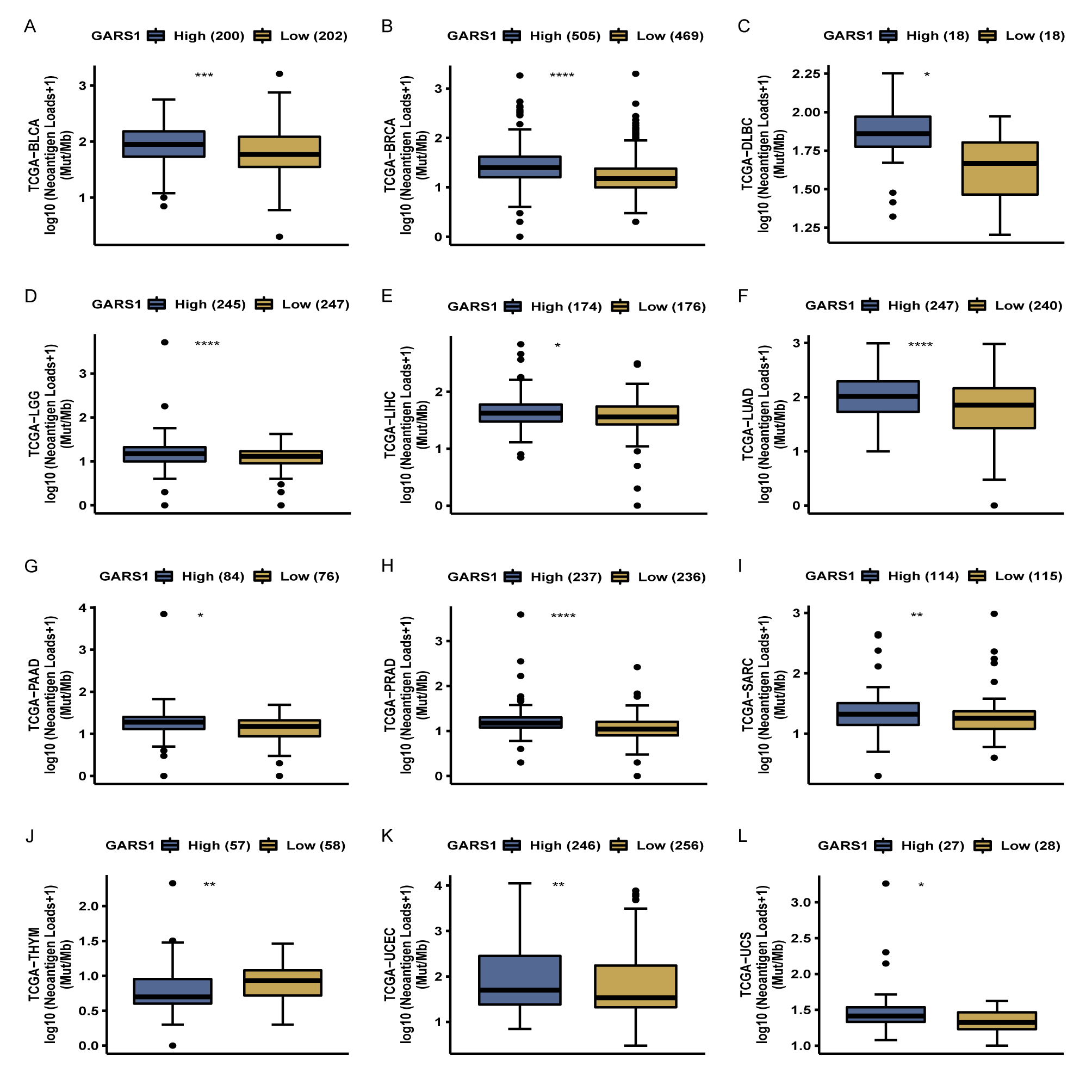

Supplement: Supplementary Figure 2 — The correlation between GARS1 expression and neoantigens in pan-cancer. [file Image_2.tif]
