# Supplementary material for: Transcriptome sequencing and single-cell sequencing analysis identify GARS1 as a potential prognostic and immunotherapeutic biomarker for multiple cancers, including bladder cancer
Source: Front Immunol. 2023 Jun 19;14:1169588. doi: 10.3389/fimmu.2023.1169588 (PMC10315539; doi:10.3389/fimmu.2023.1169588)
Supplement: Supplementary file 4 [file DataSheet_1.zip › Data Sheet 1/Data and R-Script/Paper data/Annotations.docx]

01 GARS1-DEG-TCGA+GTEx: Differential expression of GARS1 based on data after integration of TCGA and GTEx cohorts

02 GARS1-Exp-GTEx: Expression of GARS1 in normal tissues based on GTEx cohorts

03 GARS1-Exp-CCLE: Expression of GARS1 in tumor cell lines based on CCLE database.

04 GARS1-OS.cox: Correlation of GARS1 expression and OS in cancer types.

05 GARS1-DFS.cox: Correlation of GARS1 expression and DFS in cancer types.

06 GARS1-DSS.cox: Correlation of GARS1 expression and DSS in cancer types.

07 GARS1-PFS.cox: Correlation of GARS1 expression and PFS in cancer types.

08 GARS1-estimate.cor: Correlation of GARS1 expression and tumor immune microenvironment in cancer types.

09 GARS1-TIMER.cor: Correlation of GARS1 expression and immune cells in cancer types based on TIMER database.

10 GARS1-ICP.cor: Correlation of GARS1 expression and immune checkpoint genes in cancer types.

11 GARS1-MMP.cor: Correlation of GARS1 expression and mismatch repair genes in cancer types.

12 GARS1-TMB.cor: Correlation of GARS1 expression and tumor mutational burden in cancer types.

13 GARS1-MSI.cor: Correlation of GARS1 expression and microsatellite instability in cancer types.

14 GARS1-TISCH.exp(scRNA-seq): Single-cell analysis of the average expression of GARS1 in different cell types in pan-cancer base on TISCH portal.

15 GARS1-cancerSEA.cor(scRNA-seq): Single-cell analysis of the relevance of GARS1 across 14 functional states in distinct cancers based on the cancerSEA portal.

16 GARS1-CellMiner.cor: Drugs significantly related to GARS1 based on CellMiner portal.

17 GARS1-qRT-PCR-cell line: Detection of GARS1 expression in bladder cancer cells and normal uroepithelial cells SV-HC-1 by qRT-PCR

18 GARS1-qRT-PCR-Si: Detection of GARS1 knockdown efficiency by siRNA by qRT-PCR

19 GARS1_UC3-CCK8: CCK-8 test assesses the proliferative capacity of bladder cancer UC3 after knockdown of GARS1.

20 GARS1_T24-CCK8: CCK-8 test assesses the proliferative capacity of bladder cancer T24 after knockdown of GARS1.

21 Transwell result-ImageJ: Transwell migration test assesses migration of bladder cancer cells after knockdown of GARS1.

22 Wonding Heading result-ImageJ: Wound Healing test assesses migration of bladder cancer cells after knockdown of GARS1.
